# Test-retest reliability and minimal detectable change scores for the short physical performance battery, one-legged standing test and timed up and go test in patients undergoing hemodialysis

**DOI:** 10.1371/journal.pone.0201035

**Published:** 2018-08-22

**Authors:** Lucía Ortega-Pérez de Villar, Francisco José Martínez-Olmos, Anna Junqué-Jiménez, Juan José Amer-Cuenca, Javier Martínez-Gramage, Tom Mercer, Eva Segura-Ortí

**Affiliations:** 1 Department of Physiotherapy, Universidad Cardenal Herrera-CEU, CEU Universities, Valencia, Spain; 2 Nephrology Department, Hospital de Terrassa, Consorci Sanitari Terrassa, Terrassa, Spain; 3 Centre for Health, Activity and Rehabilitation Research School of Health Sciences, Queen Margaret University, Musselburgh, Edinburgh; Universita degli Studi di Perugia, ITALY

## Abstract

Functional tests are commonly used for chronic kidney disease (CKD) patients undergoing hemodialysis (HD). However, the relative and absolute reliability of such physical performance-outcome assessments must first be determined in specific patient cohorts. The aims of this study were to assess the relative and the absolute reliability of the Short Physical Performance Battery (SPPB), One-Legged Stance Test (OLST), and Timed Up and Go (TUG) test, as well as the minimal detectable change (MDC) scores for these tests in CKD patients receiving HD. Seventy-one end-stage CKD patients receiving HD therapy, aged between 21 and 90 years, participated in the study. The patients completed two testing sessions one to two weeks apart and performed by the same examiner, comprising the following tests: the SPPB (n = 65), OLST (n = 62), and TUG test (n = 66). High intraclass correlation coefficients (≥0.90) were found for all the tests, suggesting that their relative reliability is excellent. The MDC scores for the 90% confidence intervals were as follows: 1.7 points for the SPPB, 11.3 seconds for the OLST, and 2.9 seconds for the TUG test. The reliability of the SPPB, OLST, and TUG test for this sample were all considered to be acceptable. The MDC data generated by these tests can be used to monitor meaningful changes in the functional capacity of the daily living-related activity of CKD patients on HD.

## Introduction

Renal failure is a common problem with more than two million people worldwide were being treated by dialysis because of chronic kidney disease (CKD) [1]. According to the EPIRCE (Epidemiology in Chronic Renal Failure in Spain) study, 10% of the Spanish adult population suffers from some form of renal failure, with 6.8% presenting stage 3–5 CKD; in 2010, this meant that approximately 4 million people in Spain suffered from CKD requiring renal replacement treatment [2]. Hemodialysis (HD) is the most common renal replacement treatment, but other possibilities include peritoneal dialysis or kidney transplantation. The latter is especially desirable as a definitive treatment, given that patients on long-term HD have high levels of comorbidity (mainly cardiovascular problems) and physical function problems [3].

The benefits of exercise for CKD are well described in the literature, and so, since the early 80s, these patients have been prescribed exercise programs as part of their treatment. Physical function tests are commonly used to assess the effectiveness of exercise and other interventions, and these should be chosen based on their specific reliability in the CKD patient population. A previous study investigated the relative and absolute reliability and the minimal detectable change (MDC) of several physical functional tests, including the sit to stand 10 and 60, one heel rise test, handgrip test, and 6-minute walking test [4], but there are no studies regarding the reliability of other commonly used tests such as the Short Physical Performance Battery (SPPB), One-Legged Standing Test (OLST), or Timed Up and Go (TUG) test. Various authors have reported the functional properties of these tests for several sample groups, especially in elderly populations, but these tests remain insufficiently studied in CKD groups [5–18].

The SPPB is a simple test that measures lower extremity function using tasks that mimic daily activities; it is particularly useful for predicting outcomes such as falls, institutionalization, and death in elderly populations [5]. Although this test has been applied to CKD patients [6,7], neither its relative and absolute reliability nor its MDC have previously been calculated.

The OLST, also known as the one-leg stand [8,9], one-legged stance [10], single leg stance time [11,12], or unipedal balance test [13], measures the time, in seconds, that a person can stand on one leg, and is also a good predictor of falls [14]. To the best of our knowledge, no previous studies have use this test in CKD populations.

Finally, the TUG test is a simple and valid method for assessing patients’ levels of functional mobility [15]; it measures the time taken for an individual to stand up from a chair, walk three meters, turn, walk back, and sit back down. The TUG test has been used for different chronic diseases such as Alzheimer, chronic heart failure, or chronic obstructive pulmonary disease [16–18]. It has also been used in CKD patients undergoing HD [19–22] but neither its relative and absolute reliability nor its MDC have been previously calculated.

### Aims and hypothesis

The aim of this study was to calculate the test–retest reliability of the SPPB, OLST, and TUG test and to calculate their absolute reliabilities with the standard error of measurement (SEM) and MDC scores at the 90% confidence interval (MDC_90_) threshold.

## Materials and methods

### Design

This was a prospective, nonexperimental, and descriptive research study.

### Setting and participants

The participants were recruited from two HD units in Valencia and one unit in Barcelona (Spain) between 2013 and 2015. All the participants were explained the protocol and the procedures to be used, and signed their written informed consent prior to participation. This study was approved by the Ethics Committee at the *Hospital Universitario Doctor Peset* and is registered at ClinicalTrials.gov (reference number NCT02830490). The attending nephrologist reviewed and authorized their patients’ potential inclusion before the subjects were approached to solicit their interest. Patients were included in the study if they had been receiving maintenance HD for at least 3 months and did not have any acute or chronic medical conditions that would preclude the collection of the test data; they were excluded if they had recently had a myocardial infarction (within 6 weeks), unstable angina, malignant arrhythmias, or any disorder that was exacerbated by activity. The following demographic and clinical data were collected from the patients’ medical histories: age, sex, body mass index, time on HD, creatinine, albumin, and hemoglobin levels, cause of kidney disease, and the Charlson Comorbidity Index score.

### Procedure

Participants performed the SPPB, OLST, and TUG tests twice, with an interval of one to two weeks between the testing sessions (test–retest evaluation research format), always immediately before the first HD session of the week. Every effort was made to maintain consistency between the testing sessions, including control of factors such as the day of the week, time of day, testing area, and the person conducting the assessment, although not all the subjects could be assessed in both sessions. At the two HD units in Valencia, two different physical therapists (researchers 1 and 2) with 11 and 8 years’ experience in physical function evaluation, respectively, performed and assessed the tests; a renal nurse with 5 years’ experience in evaluating physical function assessed the participants at the third HD unit in Barcelona.

#### Short physical performance battery

The SPPB objectively measures lower extremity function, including performance-based balance, endurance, and strength. Each component is scored from 0 to 4 and summed to yield scores between 0 (poor) and 12 (best) performance [5] (Table 1).

**Table 1:** Short physical performance battery scoring.

*Test*		Scoring	Total
***Balance Test***	**Side by side:** the subject is asked to stand with both feet side by side and the time they can maintain the posture is measured	0 → Unable or 0–9 s 1 → 10 s	4 points
	**Semi-tandem:** The subject is asked to stand with one foot slightly in front of the other and the time is they can maintain the posture measured	0 → Unable or 0–9 s 1 → 10 s	
	**Tandem:** The subject is asked to stand with one food in front of the other and the time they can maintain the posture is measured	0 → Unable or 0–2 s 1 → 3–9 s 2 → 10 s	
***4-m gait speed***	The time taken for the subject to walk 4 m at their normal pace is measured twice; the best score from the two trials is used. Use of a walking aid in the test was recorded.	1 → ≥ 8.70 s 2 → 6.21–8.70 s 3 → 4.82–6.20 s 4 → ≤ 4.82	4 points
***STS-5***	The time taken for the subject to rise 5 times, as fast as possible, from sitting in a chair is measured. The test is completed with the patient’s arms crossed across their chest and they are not allowed to use any tools to help them to stand. The chair is armless and is situated against a wall in order to help maintain its stability and to avoid participant falls.	0 → ≥ 60 s 1 → ≥ 16.70 2 → 13.70–16.69 s 3 → 11.20–13.69 s 4 → ≤ 11.19 s	4 points

**m:** meters; **s:** seconds; **STS**: Sit to Stand

To test standing balance, the participants were asked to maintain their feet in the side-by-side, semi-tandem (heel of one foot beside the big toe of the other foot), and tandem (heel of one foot directly in front of the other foot) positions for 10 seconds each. In order to test endurance, we asked the subjects to walk for four meters at their normal pace. Participants were allowed to use their usual walking aid, although they were encouraged not to use it, and were scored according to the quartiles for the length of time required. Lower limb strength was tested by asking the subjects to fold their arms across their chests while standing up and sitting down five times (STS-5) as quickly as they could. The chair used for the test had no armrests and was backed up against a wall to minimize the risk falling. A stopwatch recorded the time taken until the peak of the fifth rise [23,24].

#### One-legged standing test

The OLST is a good predictor of falls [14]; in elderly cohorts when the maximum standing time is 30 s with open eyes, the ICC ranges from 0.60 [8] to 0.86 [11], and the MDC_95_ is 24.1 s [11]; for individuals with a hip fracture in the affected leg the ICC is 0.75 and the MDC_95_ is 10.7 s, while in the non-affected leg the ICC is 0.83 and the MDC_95_ is 5.5 s [12]; in patients with lower limb amputation the ICC is 0.87 with open eyes and using a maximum time of 60 s, and the MDC_95_ is 2.74 s [9].

To perform the OLST patients had to maintain a one-legged stance for as long as they could with their eyes open, and allowing them to freely-move their arms. All subjects wore shoes and they were allowed to choose their preferred leg; if they experienced pain or other symptoms in the first leg they were permitted to use the other leg. The participants were given three trials to try to achieve 45 seconds, and they were verbally encouraged to maintain the one-legged standing position for as long as possible during each trial; the longest balance time from the three recorded trials was used for the data analysis. The test concluded if the participant used their arms to touch the wall, if the raised foot touched the ground, if the subject moved the standing foot, or when 45 seconds had been achieved [13].

#### Timed up and go test

The TUG test has shown excellent test–retest reliability in older adults (ICC > 0.98) [15,25], chronic heart failure patients (ICC = 0.93) [16], and those with Parkinson (ICC = 0.80) [26] or Alzheimer disease (ICC = 0.985–0.988; MDC_90_ = 4.09 s) [27]. Here the TUG test subjects were given verbal instructions to stand up from a standard arm chair (using the arms if necessary), to walk three meters as quickly and safely as possible, turn back at a cone set out by the researchers, walk back, and sit down in the chair. The participants were allowed to wear their regular footwear and to use a walking aid if needed. A stopwatch was started on the word “go” and stopped when the subject was fully seated with their back against the backrest. The time to complete the test was recorded in three consecutive trials, using the first one to familiarize the subjects with the test. The best time from the three trials was analyzed [25,28,29].

#### Human activity profile

To evaluate the physical activity level, the participants were asked to complete the Human Activity Profile (HAP) that has been validated in the population with renal disease [30]. The HAP questionnaire consists of a list of 94 items, which assesses activities ranked in ascending order of level of energy. The participants had three possibilities to answer: (1) still doing this activity, (2) have stopped doing this activity, or (3) never did this activity. The HAP assesses the Maximal activity score level of activity (MAS) (the highest level of activity) and the adjusted activity score (ASS). The MAS is calculated as the activity with the highest oxygen consumption requirement that the subject still performs, while the ASS = MAS—number of less demanding activities the subject has stopped performing. The ASS gives us a better estimate of the range activities performed and of the presence of impairment. Depending on the AAS, subjects can be classified as impaired activity (AAS less than 53), moderately active (AAS 53–74) or active (AAS greater than 74) [31]. This questionnaire has been shown to be test-retest reliability in this population, being the ICC for the MAS = 0.76 (95% confidence interval = 0.53–0.89) and the MDC_95_ 15.1 points, while for ASS the ICC was = 0.92 (95% confidence interval = 0.83–0.97), being the MDC_95_ 11.4 points [32].

### Statistics

Normally-distributed descriptive data are reported as the mean plus the standard deviation (SD), or otherwise, as the median plus the range. The Kolmogorov–Smirnov test was used to assess the normality of the data. We also performed paired comparisons with the paired t-test or the Wilcoxon signed rank test to assess any systematic bias between the trials. The ICC (model alpha) and a two-way random-effects model were used to assess the test–retest reliability of the data for all the repeated tests; we considered an ICC above 0.75 to demonstrate good reliability, although for clinical measurements it has been suggested that the ICC should exceed 0.90 [32]. The SEM was used to determine the absolute reliability of the tests and represents the extent to which the outcome can vary in the measurement process. It was calculated with the following formula:

$$\mathrm{SEM}=\mathrm{SD}\times \sqrt{\left(1-r\right)}$$

Where r is the ICC for the participant groups.

The MDC is defined as the amount of change in a measurement required to conclude that the difference is not attributable to error; it is the smallest change that falls outside the expected range of error thus, any change exceeding the MDC_90_ is considered genuine and indicates confidence in the test’s predictive abilities [4,27,33,34]. The MDC_90_ was computed from the SEM with the following formula:

$${\mathrm{MDC}}_{90}=\mathrm{SEM}\times 1.65\times \sqrt{2}$$

A Bland-Altman plot of each participant’s mean score (SPPB, OLST, TUG) plotted against their difference score (trial 1-trial 2) was constructed to display the spread of difference scores about the mean difference score. The Bland-Altman plots also display the 95% limits of agreement (95% LOA) which represents the expected range of difference scores across trials of the tests. The 95% LOA was calculated as the difference in mean scores of the tests ± SD x 1.96, with the SD as the standard deviation of the difference scores.

Correlation between the three tests and hemoglobin, albumin and creatinine was explored thouth the Spearman correlation coefficient.

We set the level of significance required to a probability of *P* ≤ 0.05 for all our statistical analyses. The data were managed and analyzed using the Statistical Package for Social Sciences (SPSS) version 20.0 for Windows.

## Results

**Fig 1:**
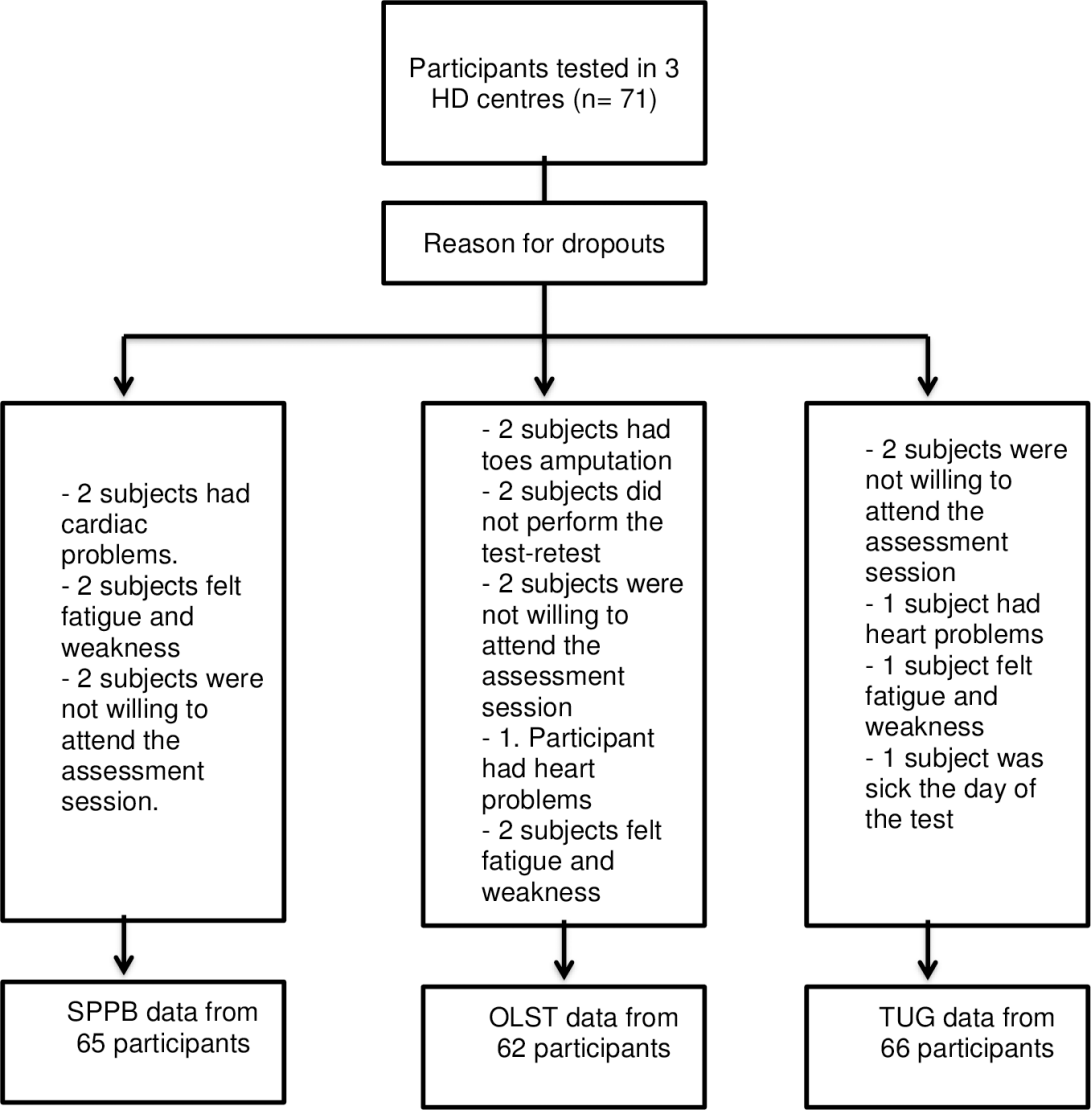
Flow chart for study participants in the test—retest reliability and minimal detectable change for the SPPB, the OLST and TUG HD: Hemodialysis; OLST: One-Legged Standing Test; SPPB: Short Physical Performance Battery; TUG: Timed Up and Go test.

Data were collected from 71 participants (29 women and 42 men) with end-stage CKD receiving HD treatment at three different HD units; the mean age was 61.7±16.4 years. Some demographic details were unavailable (e.g., no height for one participant); descriptive statistics for all the participants are shown in Table 2. The activity level of the sample according to the human activity profile adjusted activity score was low, with a mean score below 53. Fig 1. shows the number of subjects who performed each test; there were 6, 9, and 5 drop outs for the SPPB, OLST, and TUG test, respectively, and the reasons for these withdrawals are shown in Fig 1; no adverse events occurred during testing.

**Table 2:** Demographic and clinical data for the participants in the test–retest reliability and minimal detectable change study (N = 71).

*Characteristic*	Value
***Age*, *mean (SD)***	61.7 (16.4)
***Sex (women*:*men)***	29:42
***Body Mass Index (kg/m***^***2***^***)*, *mean (SD)***	25.87 (6.23)
***Time on hemodialysis (median in months; P25–P75)***	56 months 34–96 months
***Creatinine levels (mg/dL)*, *mean (SD)***	9.23 (3.04)
***Hemoglobin levels (g/dL)*, *mean (SD)***	11.00 (1.37)
***Albumin levels (g/dL)*, *mean (SD)***	4.18 (4.52)
***Cause of Kidney Disease (number of participants)***	
• ***Diabetes Mellitus***	14
• ***Glomerulonephritis***	13
• ***Nephroangiosclerosis***	6
• ***Lupus***	3
• ***Pyelonephritis***	5
• ***Polycystic kidney disease***	3
• ***High Blood Pressure***	4
• ***Others***	23
***Charlson Comorbidity Index*, *mean (SD)***	6.73 (2.43)
***Physical Activity Level (Human Activity Profile–Adjusted Activity Score)*, *mean (SD)***	38.6 (32.0)

**SD**: standard deviation

The results of the repeated tests are shown in Table 3 (see S1 Table Original data from SPPB, S2 Table Original data from OLST, S3 Table Original data from TUG). For the SPPB, the mean plus SD in trial 1 and trial 2 were 9.6±3 and 10±2.9 repetitions, respectively (*p* = 0.94); for the OLST it was 13.5±14.9 s for trial 1 and 15.1±15 s for trial 2 (*p* = 0.89); and for the TUG test it was 11.2±6.3 s and 10.7±5.8 s for trial 1 and 2, respectively (*p* = 0.96). The ICCs were high for all of the outcome measurements: 0.94 (95% confidence interval [CI] = 0.91–0.97) for the SPPB; 0.90 (95% CI = 0.83–0.94) for the OLST, and 0.96 (95% CI = 0.94–0.98) for the TUG test. The paired comparisons showed insignificant differences between trial 1 and trial 2 for all three tests. Table 4 shows the MDC_90_ values for the SPPB, OLST, and TUG test (1.7 points, 11.3 s, and 2.9 s, respectively).

**Table 3:** Reliability results for the SPPB, OLST, and TUG physical performance tests in patients undergoing hemodialysis.

*Test*	No. of participants	Trial 1	Trial 2	ICC for trial 1 vs. trial 2	95% CI for ICC	*P* of a significant difference between trial 1 and trial 2
		Median (Min-Max)	Median (Min-Max)			
***SPPB (points)***	65	11 (0–12)	11(0–12)	0.94	0.91–0.97	0.942
***OLST*** ***(seconds)***	62	4.4 (0–45)	8.1 (0–45)	0.90	0.83–0.94	0.895
***TUG test*** ***(seconds)***	66	9.0 (4.60–37.5)	8.6 (3.72–32.5)	0.96	0.94–0.98	0.962

**ICC:** intraclass correlation coefficient; **CI:** confidence interval; **OLST:** One-Legged Standing Test; **SPPB:** Short Physical Performance Test; **TUG:** Timed Up and Go

**Table 4:** Standard error of measurement for repeated measures and minimal detectable change scores at a 90% confidence interval (MDC_90_) for the SPPB, OLST, and TUG test.

*Test*	MDC_90_	CI 95%	SEM	CI 95%
***SPPB*** ***(points)***	1.7	1.3–2.1	0.72	0.56–0.91
***OLST*** ***(seconds)***	11.3	8.9–14.2	4.82	3.80–6.10
***TUG*** ***(seconds)***	2.9	2.2–3.7	1.24	0.96–3.66

**CI:** confidence interval; **OLST**: One-Legged Standing Test; **SPPB**: Short Physical Performance Battery; **TUG**: Timed Up and Go test; **SEM**: standard error of measurement.

**Fig 2:**
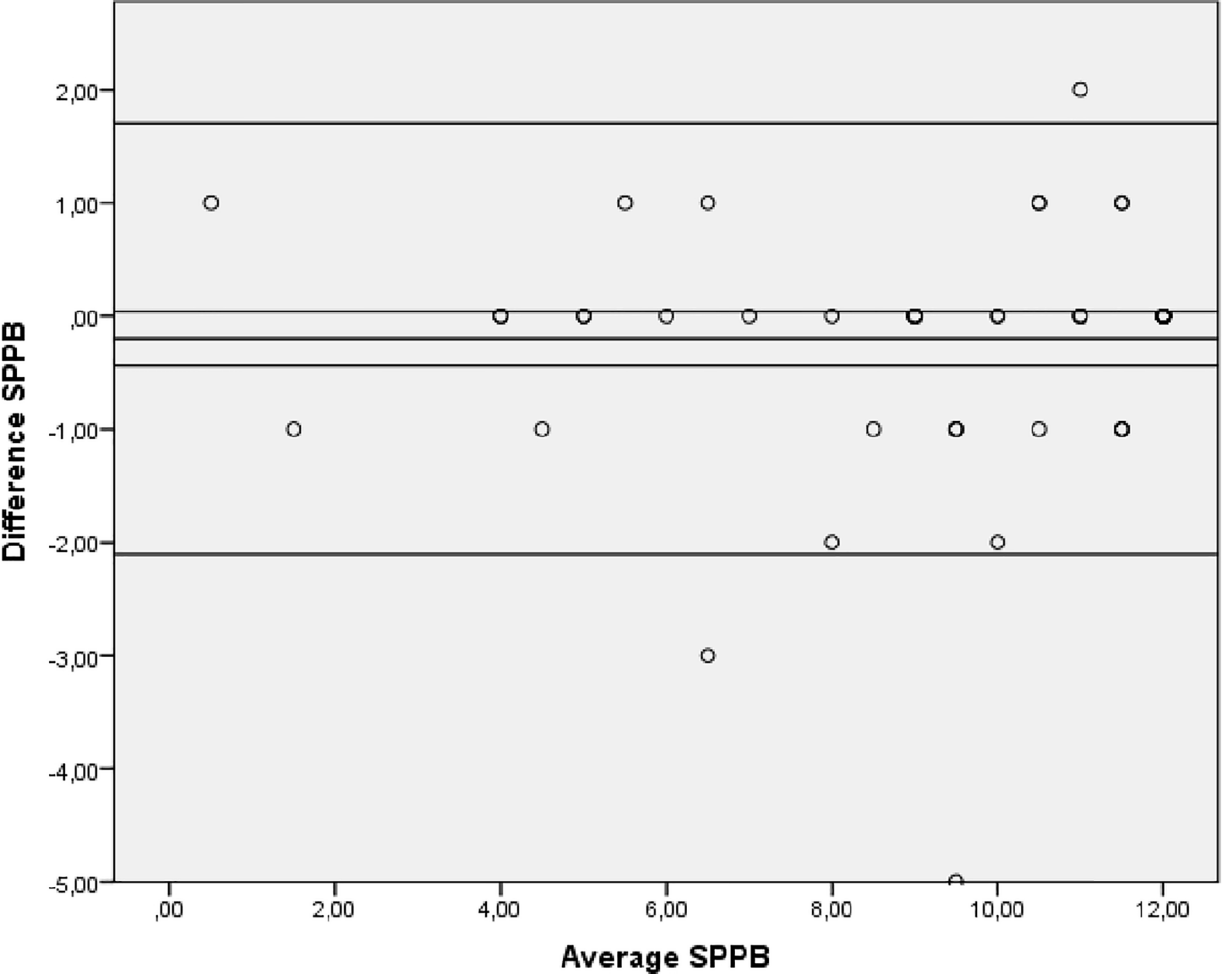
Bland-Altman plot showing levels of agreement for the test-retest data for the SPPB. SPPB: Short Physical Performance Battery.

**Fig 3:**
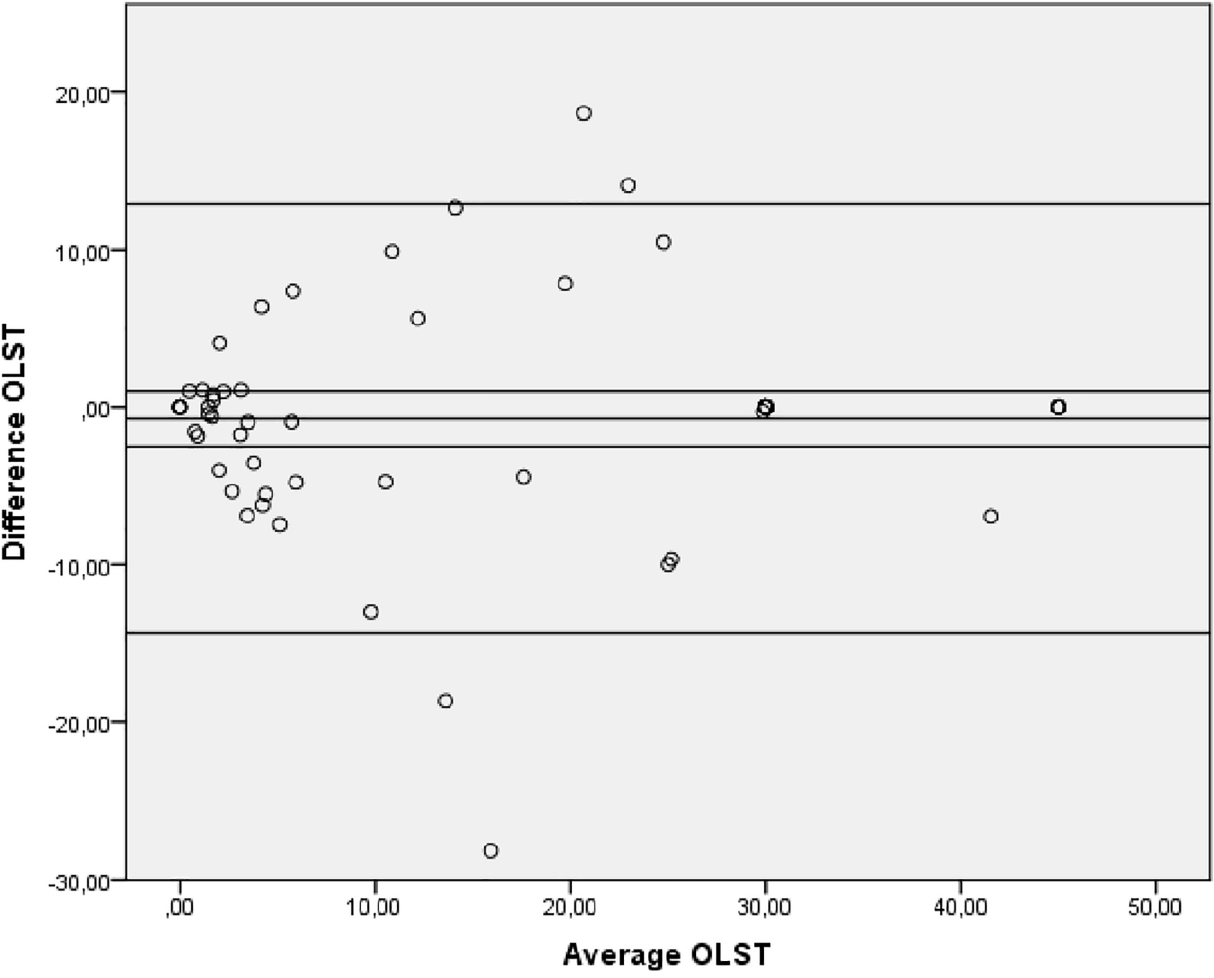
Bland-Altman plot showing levels of agreement for the test-retest data for the OLST. OLST: One-Legged Standing Test.

**Fig 4:**
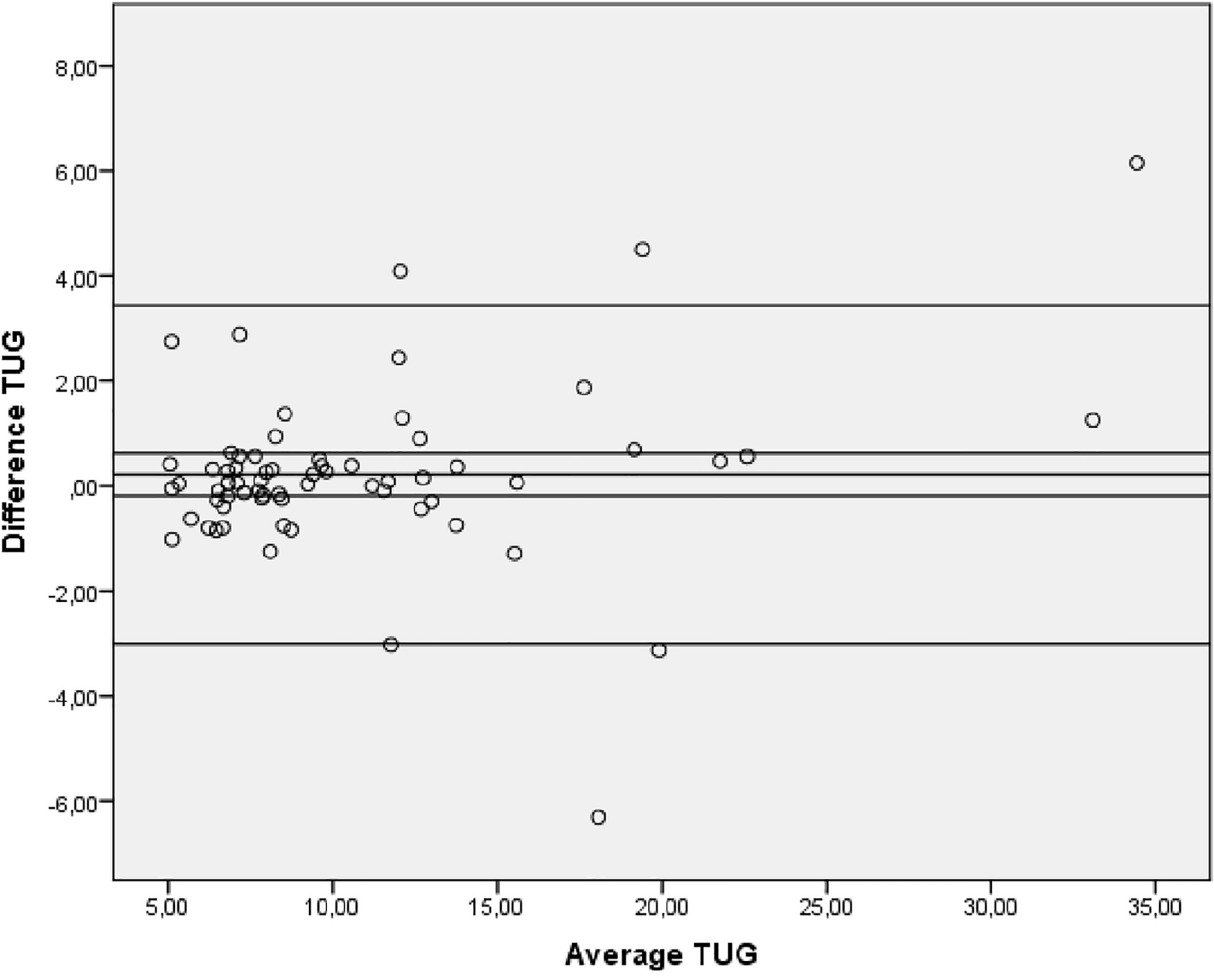
Bland-Altman plot showing levels of agreement for the test-retest data for the TUG. TUG: Timed Up and Go test.

Bland-Altman plots indicated no systematic bias as scores were distributed above and below the mean difference (Fig 2, Fig 3 and Fig 4).

Spearman correlation coefficient showed a significant correlation only between the TUG and the inverse creatinine value (r = 0.375; p = 0.004)

## Discussion

The SPPB, OLST, and TUG test are widely used performance tests, probably owing to their simplicity and low cost. Our findings demonstrated that the test–retest relative reliability (ICC) for the use of these clinical tests for CKD patients was excellent: all three values reached or exceeded 0.90±33, meaning that the two successive assessments we performed one to two weeks apart were very reproducible.

### Test–retest reliability

The SPPB examines three areas of lower-extremity function (static balance, gait speed, and getting in and out of a chair) that are representative of essential tasks for independent living among CKD patients on HD. The SPPB is useful for predicting outcomes such as falls, institutionalization, and death in elderly population [5], and although it has previously been applied to HD patients [6,35], to our knowledge, ours is the first study describing the relative reliability of the SPPB in patients undergoing HD. Our results showed that this test has excellent test–retest reliability (ICC = 0.94; 95% CI = 0.91–0.97), and are consistent with values reported for a community-dwelling older population (ICC = 0.82; n = 487; mean age 74.1±5.7 years) [36] and for older women (ICC = 0.88–0.92; n = 1002; mean age 78.3±0.3 years) [37].

Similar to our study, Studenski et al. [36] performed the test–retest after one week, although they used a different testing site between trials: first during an outpatient clinic visit and then as part of a comprehensive home visit. In our case, we acquired all the measurements for both trails at the same location and within one or two weeks. In our study the ICC for the SPPB was high, suggesting that it is a good physical performance test for identifying loss of mobility in CKD patients undergoing HD. Future longitudinal studies should clarify whether the SPPB can predict difficulties in the activities of daily living in HD patients, as it can in elderly and older hospitalized patient populations [37,38].

No previous studies have reported the relative reliability for the OLST in patients undergoing HD, although the OLST ICC values reported in other populations are generally lower than our results (ICC≥0.90). In elderly populations the ICC ranges from 0.60 [8] to 0.86 [11], following hip fracture it was 0.75 and 0.83 in the affected and non-affected leg, respectively [12], and it was 0.87 for patients with a lower-limb amputation [9]. In contrast, an ICC of 0.994 was reported for a subgroup of 50 healthy military health-care beneficiaries aged 18 and older.

There are a wide variety of published protocols for performing the OLST, but surprisingly little consensus regarding how it should be conducted. For example, some studies use a maximum time of 10 seconds [39,40], and others 30 seconds [8,12,41], 45 seconds [13,39], or 60 seconds [9,11,42]. We chose to use 45 seconds as maximum time because Briggs et al. [10] posit that a limit of 45 seconds results in normal data distribution [10,13]. Another variable is the number of attempts the patient is allowed to achieve the maximum time: while some studies do not report this factor [8,12], in other trials it ranges between three [39,41,42] and five [9,11]. Additionally, some authors use the average of the trials for their statistical analyses [11,39] while others use the single longest time achieved [9,10,42]. Following the procedure published by Hurvitz et al. [13], itself based on Briggs et al. [10], we performed three trials and used the longest time achieved for our data analysis. This strategy appears to provide a good indication of balance capabilities because the best trial results were almost always obtained among the first three test trial results [10,13].

The details of how the OLST studies are executed also often differ: as in other studies we allowed our participants to keep their eyes open [9,39,41,42], wear shoes, choose the leg they preferred for the test, and to move their arms to help maintain their balance [13]. Moreover, our sample size was larger than that of previous studies (n = 62) and the ages included ranged from 21 to 90 years (mean 61.4±16.4 years), making ours a relatively young sample compared to other studies (see Table 5). Future studies should aim to assess if the OLST is useful for predicting falls in CKD patients.

**Table 5:** Characteristics of selected studies which use the one-legged standing test.

Author	Type of population	N	Range age min-max	Mean age (SD)
**Wolinsky et al. [8]**	Elderly African Americans	53	50–64	56.6
**Sherrington & Lord [12]**	Hip fracture	30	62–95	79.8 (10.0)
**Goldberg et al. [11]**	Older community-dwelling individuals	25	60–89	72.0 (9.1)
**Kristensen et al. [9]**	Lower-limb amputation	36		67.4 (10.6)
**Giorgetti et al. [42]**	Non-disabled community	21	69–85	73.1
	Older people with some physical disability	21	61–89	75
**Chomiack et al. [43]**	Parkinson disease	27		67.1 (10.2)

**SD**: standard deviation

The TUG test is a validated and commonly used method for assessing functional mobility; its relative reliability values have been reported in different populations including elderly (ICC = 0.98–0.99) [15,25], chronic heart failure (ICC = 0.93) [16], Parkinson disease (ICC = 0.80) [26], and Alzheimer disease (ICC = 0.985–0.988) [27] cohorts. Our results showed that the relative reliability of this test for patients undergoing HD is excellent (ICC = 0.96), therefore suggesting that this is an appropriate test for assessing this aspect of physical function in CKD patient groups. Additionally, this was the only test that correlated with the inverse creatinine values of the sample.

Taken together, our findings demonstrate that test–retest reliability for the SPPB, OLST, and TUG clinical tests was excellent. Factors that might explain these good results, and that should therefore be considered in the application these tests in clinical environments, include performing these tests (i) before a HD session, (ii) on the same day of the week, and (iii) after adequate research training and standardization of the assessors’ instructions. However, it is surprising that the relative reliability (ICC) in a sample with such high comorbidity (CKD patients on maintenance HD) was higher than in other cohorts with, presumably, lower health status variability (e.g. elderly populations with no chronic disease). This could mean that young people receiving renal replacement treatment are usually in a better physical condition than elderly populations receiving HD, leading to the increased consistency seen in the former in this present study.

Another reason could be the uniformity of our protocol which we designed to ensure standardization, both of the procedures and between the researchers performing the tests. Our testing instructions were the result of a consensus between the different research teams at each center undertaking the study. Surprisingly, our review of previously published studies regarding functional testing, revealed inconsistencies between the testing protocols used across a variety of tests, including the OLST. These factors might lead to inappropriate results being reported and may hinder meaningful comparison between the outcomes of different studies. Thus, we believe it is very important that both researchers and clinicians assess physical functioning in future studies using the same tools and by implementing standardized instructions.

### Minimal detectable change

Despite the excellent test–retest reliability results for our patient cohort, the performance of individual participants between sessions still substantially varied, producing high MDC values (Table 4). The MDC_90_ is the threshold of change that a measurement must reach in order to exceed the anticipated measurement error and variability, and is a conservative estimate of clinically meaningful score changes. In this case, the magnitude of clinically meaningful change in these physical performance tests can help clinicians and researchers to identify important functional changes in CKD patients undergoing HD [4]. The MDC for the SPPB, OLST, and TUG test have been previously studied in other populations including the elderly [5,8,11,43], people recovering from a hip fracture [12] or lower-limb amputation [9], and in groups with Alzheimer disease [27]. Nevertheless, to our knowledge, this is the first study to calculate the MDC of these tests in patients with CKD undergoing HD.

Our results produced an MDC_90_ of 1.7 points for the SPPB, whereas in an elderly population, a change of one point was representative of a meaningful difference in the risk of future mortality and the incidence of disability [5]. Another large study of older adults (n = 482; mean age 74.1±5.7 years) reported a SEM of 1.42 points [44], compared to the SEM of 0.72 points we obtained in this study. In this case, the time frame of the test–retest assessment was longer than in our study: the subjects were evaluated at the participant’s house every three months for the first year and every 6 months for the second year. In our study we strictly replicated all the measurement conditions, but even so, the physiological and clinical status of patients undergoing HD can widely vary, potentially leading to heterogeneity in the results.

Our OLST results gave an MDC_90_ of 11.3 s, whereas in a community-dwelling population, the MDC_95_ was 24.1 s [11]. This, perhaps surprising difference can be explained by the high SD in the latter study sample (20.4 s) [45]. In patients with a lower-limb amputation the MDC_95_ was 2.74 s [9], and this difference can also be related to the evaluation procedure: while we performed three trials with a maximum time of 45 seconds, other studies performed five trials with a maximum time of 60 seconds [9,11]. We chose three rather than five trials to try to achieve the longest time possible (in the knowledge that the best score is usually obtained in the first three trials), while also aiming to reduce variability and to avoid muscle fatigue [10].

The MDC_90_ for the TUG test in this present study was 2.9 s. In comparison, the MDC_95_ in a cohort with Parkinson disease was 3.5 s [26] (similar to our results if we calculate the MDC_90_) and in another sample with Alzheimer disease, the MDC_90_ was 4.09 s [27]. The high MDC found in the Alzheimer disease study can be explained by its high SD (19.95 ± 9.81 s in mild-moderate disease and 28.01 ± 17.49 s in moderate-severe to severe disease); patients with a higher level of dementia produce more variable results and need more time to perform the test compared to less demented subjects, thus generating higher MDC scores. Another important difference is the number of trials performed: while we carried out three trials, Ries at al. [27] performed two trials in patients with Alzheimer disease and Huang et al. [26] only measured the TUG once, so as to avoid fatigue (although they concluded that more trials would increase the stability of the measurement and would reduce its MDC). Hence, performing more than one trial increases the stability of the test, and as a result, decreases the MDC.

In summary, the MDC_90_ results that we obtained in this study (1.7 points for the SPPB, 11.3 s for the OLST, and 2.9 s for the TUG test) represent the threshold-change values required to be 90% certain that any changes noted in the test results for any given individual patient are not due to internal variability. In the clinical field, researchers and clinicians should use these MDC values to determine whether differences in the test results obtained between follow-up trails in their CKD patients on maintenance HD represent true changes which may be associated with poor prognosis.

### Study limitations

The main limitation of this study was the variability of our cohort in terms of its broad sample age range which may have introduced error related to the probable increased presence of comorbidities in older patients. It is also worth noting that the patient participation rate was low. Additionally, we did not register interdialytic weight gain between the first and the second evaluation day, though we tried to keep all other factors stable (HD session of the week, time, assessor). Moreover, only 30 minutes were available to perform these assessment tests before the HD session started which may have led us to rush in some cases. However, despite this time constraint, we tried to limit extrinsic variation by following a strict methodology. Another potential limitation to inter-study comparisons is the lack of academic consensus on the exact OLST testing procedure.

## Conclusions

In conclusion, our results demonstrate excellent test–retest reliability for the SPPB, the OLST, and the TUG test in CKD patients undergoing HD. The MDC_90_ values for each test provide clinicians with useful threshold values for identifying true changes beyond those that can be expected from individual variability. This information will help care givers to monitor changes in the performance of their patients over time and to assess the effectiveness of interventions to maintain or improve the physical performance of patients receiving HD treatment.

## Supporting information

S1 Table Original data from SPPB. (XLSX)

S2 Table Original data from OLST. (XLSX)

S3 Table Original data from TUG. (XLSX)

## References

[1] Brück K, Stel VS, Fraser S, De Goeij MC, Caskey F, Abu-Hanna A, et al Translational research in nephrology: chronic kidney disease prevention and public health. Clinical kidney journal 2015:sfv082. 10.1093/ckj/sfv082 PMC4655791 26613019

[2] Otero A, de Francisco A, Gayoso P, Garcia F, EPIRCE Study Group. Prevalence of chronic renal disease in Spain: results of the EPIRCE study. Nefrologia 2010;30(1):78–86. 10.3265/Nefrologia.pre2009.Dic.5732 20038967

[3] Odden MC, Whooley MA, Shlipak MG. Association of chronic kidney disease and anemia with physical capacity: the heart and soul study. J Am Soc Nephrol 2004 11;15(11):2908–2915. 10.1097/01.ASN.0000143743.78092.E3 15504944 PMC2776664

[4] Segura-Orti E, Martinez-Olmos FJ. Test-retest reliability and minimal detectable change scores for sit-to-stand-to-sit tests, the six-minute walk test, the one-leg heel-rise test, and handgrip strength in people undergoing hemodialysis. Phys Ther 2011 8;91(8):1244–1252. 10.2522/ptj.20100141 21719637

[5] Guralnik JM, Winograd C. Physical performance measures in the assessment of older persons. Aging Clinical and Experimental Research 1994;6(5):303–305. 10.1007/BF03324256 7893776

[6] Chen JL, Godfrey S, Ng TT, Moorthi R, Liangos O, Ruthazer R, et al Effect of intra-dialytic, low-intensity strength training on functional capacity in adult haemodialysis patients: a randomized pilot trial. Nephrol Dial Transplant 2010 6;25(6):1936–1943. 10.1093/ndt/gfp739 20100734 PMC2902890

[7] Saitoh M, Itoh H, Morotomi N, Ozawa T, Ishii N, Uewaki R, et al Impact of chronic kidney disease and anemia on physical function in patients with chronic heart failure. Cardiorenal Med 2014 8;4(2):73–81. 10.1159/000362252 25254028 PMC4164108

[8] Wolinsky FD, Miller DK, Andresen EM, Malmstrom TK, Miller JP. Reproducibility of physical performance and physiologic assessments. J Aging Health 2005 4;17(2):111–124. 10.1177/0898264304272784 15750047

[9] Kristensen MT, Nielsen AØ, Topp UM, Jakobsen B, Nielsen KJ, Juul-Larsen HG, et al Number of test trials needed for performance stability and interrater reliability of the one leg stand test in patients with a major non-traumatic lower limb amputation. Gait Posture 2014;39(1):424–429. 10.1016/j.gaitpost.2013.08.017 24021523

[10] Briggs RC, Gossman MR, Birch R, Drews JE, Shaddeau SA. Balance performance among noninstitutionalized elderly women. Phys Ther 1989 9;69(9):748–756. 2772037 10.1093/ptj/69.9.748

[11] Goldberg A, Casby A, Wasielewski M. Minimum detectable change for single-leg-stance-time in older adults. Gait Posture 2011;33(4):737–739. 10.1016/j.gaitpost.2011.02.020 21444208

[12] Sherrington C, Lord SR. Reliability of simple portable tests of physical performance in older people after hip fracture. Clin Rehabil 2005 8;19(5):496–504. 10.1191/0269215505cr833oa 16119405

[13] Hurvitz EA, Richardson JK, Werner RA. Unipedal stance testing in the assessment of peripheral neuropathy. Arch Phys Med Rehabil 2001 2;82(2):198–204. 10.1053/apmr.2001.17830 11239310

[14] Lundin H, Sääf M, Strender L, Nyren S, Johansson S, Salminen H. One-leg standing time and hip-fracture prediction. Osteoporosis Int 2014;25(4):1305–1311. 10.1007/s00198-013-2593-1 24562837

[15] Podsiadlo D, Richardson S. The timed “Up & Go”: a test of basic functional mobility for frail elderly persons. J Am Geriatr Soc 1991;39(2):142–148. 1991946 10.1111/j.1532-5415.1991.tb01616.x

[16] Hwang R, Morris NR, Mandrusiak A, Mudge A, Suna J, Adsett J, et al Timed up and go test: a reliable and valid test in patients with chronic heart failure. J Card Fail 2015. 10.1016/j.cardfail.2015.09.018 26456063

[17] Beauchamp MK, O'Hoski S, Goldstein RS, Brooks D. Effect of pulmonary rehabilitation on balance in persons with chronic obstructive pulmonary disease. Arch Phys Med Rehabil 2010;91(9):1460–1465. 10.1016/j.apmr.2010.06.021 20801268

[18] Gras LZ, Kanaan SF, McDowd JM, Colgrove YM, Burns J, Pohl PS. Balance and gait of adults with very mild Alzheimer disease. J Geriatr Phys Ther 2015 Jan-Mar;38(1):1–7. 10.1519/JPT.0000000000000020 24755691 PMC4632639

[19] Anding K, Bär T, Trojniak-Hennig J, Kuchinke S, Krause R, Rost JM, et al A structured exercise programme during haemodialysis for patients with chronic kidney disease: clinical benefit and long-term adherence. BMJ open 2015;5(8):e008709 10.1136/bmjopen-2015-008709 26316654 PMC4554901

[20] Cook SA, MacLaughlin H, Macdougall IC. A structured weight management programme can achieve improved functional ability and significant weight loss in obese patients with chronic kidney disease. Nephrol Dial Transplant 2008 1;23(1):263–268. 10.1093/ndt/gfm511 17977872

[21] Greenwood SA, Lindup H, Taylor K, Koufaki P, Rush R, Macdougall IC, et al Evaluation of a pragmatic exercise rehabilitation programme in chronic kidney disease. Nephrol Dial Transplant 2012 10;27 Suppl 3:iii126–34. 22785111 10.1093/ndt/gfs272

[22] Ling KW, Wong FS, Chan WK, Chan SY, Chan EP, Cheng YL, et al Effect of a home exercise program based on tai chi in patients with end-stage renal disease. Perit Dial Int 2003 12;23 Suppl 2:S99–S103. 17986569

[23] Guralnik JM, Ferrucci L, Simonsick EM, Salive ME, Wallace RB. Lower-extremity function in persons over the age of 70 years as a predictor of subsequent disability. N Engl J Med 1995;332(9):556–562. 10.1056/NEJM199503023320902 7838189 PMC9828188

[24] Cho H, Sohng K. The effect of a virtual reality exercise program on physical fitness, body composition, and fatigue in hemodialysis patients. Journal of physical therapy science 2014;26(10):1661–1665. 10.1589/jpts.26.1661 25364137 PMC4210422

[25] Shumway-Cook A, Brauer S, Woollacott M. Predicting the probability for falls in community-dwelling older adults using the Timed Up & Go Test. Phys Ther 2000 9;80(9):896–903. 10960937

[26] Huang SL, Hsieh CL, Wu RM, Tai CH, Lin CH, Lu WS. Minimal detectable change of the timed "up & go" test and the dynamic gait index in people with Parkinson disease. Phys Ther 2011 1;91(1):114–121. 10.2522/ptj.20090126 20947672

[27] Ries JD, Echternach JL, Nof L, Gagnon Blodgett M. Test-retest reliability and minimal detectable change scores for the timed "up & go" test, the six-minute walk test, and gait speed in people with Alzheimer disease. Phys Ther 2009 6;89(6):569–579. 10.2522/ptj.20080258 19389792

[28] Maanum G, Jahnsen R, Froslie KF, Larsen KL, Keller A. Walking ability and predictors of performance on the 6-minute walk test in adults with spastic cerebral palsy. Dev Med Child Neurol 2010 6;52(6):e126–32. 10.1111/j.1469-8749.2010.03614.x 20163429

[29] Kovacs E, Sztruhar Jonasne I, Karoczi CK, Korpos A, Gondos T. Effects of a multimodal exercise program on balance, functional mobility and fall risk in older adults with cognitive impairment: a randomized controlled single-blind study. Eur J Phys Rehabil Med 2013 10;49(5):639–648. 23820879

[30] Johansen KL, Painter P, Kent-Braun JA, Ng AV, Carey S, Da Silva M, et al Validation of questionnaires to estimate physical activity and functioning in end-stage renal disease. Kidney Int 2001 3;59(3):1121–1127. 10.1046/j.1523-1755.2001.0590031121.x 11231369

[31] Fix AJ, Daughton D. Human activity profile: professional manual: Psychological Assessment Resources; 1988.

[32] Overend T, Anderson C, Sawant A, Perryman B, Locking-Cusolito H. Relative and absolute reliability of physical function measures in people with end-stage renal disease. Physiotherapy Canada 2010;62(2):122–128. 10.3138/physio.62.2.122 21359043 PMC2871020

[33] Portney LG, Watkins MP. Foundations of clinical research: applications to practice: FA Davis; 2015.

[34] Steffen TM, Hacker TA, Mollinger L. Age- and gender-related test performance in community-dwelling elderly people: Six-Minute Walk Test, Berg Balance Scale, Timed Up & Go Test, and gait speeds. Phys Ther 2002 2;82(2):128–137. 11856064 10.1093/ptj/82.2.128

[35] Mangione KK, Craik RL, McCormick AA, Blevins HL, White MB, Sullivan-Marx EM, et al Detectable changes in physical performance measures in elderly African Americans. Phys Ther 2010 6;90(6):921–927. 10.2522/ptj.20090363 20395305

[36] Kaysen GA, Larive B, Painter P, Craig A, Lindsay RM, Rocco MV, et al Baseline physical performance, health, and functioning of participants in the Frequent Hemodialysis Network (FHN) trial. American Journal of Kidney Diseases 2011;57(1):101–112. 10.1053/j.ajkd.2010.08.021 21184919 PMC3073398

[37] Studenski S, Perera S, Wallace D, Chandler JM, Duncan PW, Rooney E, et al Physical performance measures in the clinical setting. J Am Geriatr Soc 2003;51(3):314–322. 12588574 10.1046/j.1532-5415.2003.51104.x

[38] Ostir GV, Volpato S, Fried LP, Chaves P, Guralnik JM. Reliability and sensitivity to change assessed for a summary measure of lower body function: results from the Women's Health and Aging Study. J Clin Epidemiol 2002;55(9):916–921. 12393080 10.1016/s0895-4356(02)00436-5

[39] Guralnik JM, Ferrucci L, Pieper CF, Leveille SG, Markides KS, Ostir GV, et al Lower extremity function and subsequent disability: consistency across studies, predictive models, and value of gait speed alone compared with the short physical performance battery. J Gerontol A Biol Sci Med Sci 2000 4;55(4):M221–31. 10811152 10.1093/gerona/55.4.m221 PMC12149745

[40] Springer BA, Marin R, Cyhan T, Roberts H, Gill NW. Normative values for the unipedal stance test with eyes open and closed. J Geriatr Phys Ther 2007;30(1):8–15. 19839175 10.1519/00139143-200704000-00003

[41] Bohannon RW. Single Limb Stance Times: A Descriptive Meta‐Analysis of Data From Individuals at Least 60 Years of Age. Topics in Geriatric Rehabilitation 2006;22(1):70–77.

[42] Giorgetti MM, Harris BA, Jette A. Reliability of clinical balance outcome measures in the elderly. Physiotherapy Research International 1998;3(4):274–283. 9859135 10.1002/pri.150

[43] Chomiak T, Pereira FV, Hu B. The Single-leg-stance test in Parkinson’s disease. Journal of clinical medicine research 2014;7(3):182–185. 10.14740/jocmr1878w 25584104 PMC4285065

[44] Perera S, Mody SH, Woodman RC, Studenski SA. Meaningful change and responsiveness in common physical performance measures in older adults. J Am Geriatr Soc 2006;54(5):743–749. 10.1111/j.1532-5415.2006.00701.x 16696738

[45] Bohannon RW. Responsiveness of the single-limb stance test. Gait Posture 2012;35(1):173 10.1016/j.gaitpost.2011.07.015 21890363

